# Forecasting Stalking Recidivism Using the Guidelines for Stalking Assessment and Management (SAM)

**DOI:** 10.1177/10731911221086050

**Published:** 2022-04-18

**Authors:** Sarah H. Coupland, Jennifer E. Storey, P. Randall Kropp, Stephen D. Hart

**Affiliations:** 1 Simon Fraser University, Burnaby, British Columbia, Canada; 2 University of Kent, Canterbury, UK; 3 Forensic Psychiatric Services Commission, Vancouver, British Columbia, Canada; 4 University of Bergen, Norway

**Keywords:** stalking, violence risk assessment, Guidelines for Stalking Assessment and Management, structured professional judgment

## Abstract

We examined the long-term risk for stalking recidivism and the predictive validity of ratings made using the Guidelines for Stalking Assessment and Management (SAM) in 100 stalking offenders from a forensic clinic. Overall, 45 offenders were convicted of, charged with, or the subject of police investigation for stalking-related offenses during a potential time at risk that averaged 13.47 years. Survival analyses using the Cox proportional hazards model indicated that a composite score of the presence of SAM risk factors was significantly predictive of recidivism and had significant incremental validity relative to total scores on two scales commonly used in violence risk assessment, the Screening Version of the Hare Psychopathy Checklist–Revised (PCL:SV) and the Violence Risk Appraisal Guide (VRAG). Overall ratings of risk made using the SAM, however, were not significantly predictive of recidivism. We discuss the potential uses of the SAM in stalking risk assessment and provide recommendations for future research.

Stalking is a form of violence comprising a pattern of unwanted communication or contact directed toward one or more specific victims that intentionally or recklessly causes them to reasonably fear for their own safety or the safety of others known to them (Beatty, 2003; Owens, 2016). The consequences of stalking may include serious psychological harm to victims in the form of intimidation, fear, and disruption of activities of daily living; in some cases, it may escalate to actual or attempted physical harm, up to and including lethal or life-threatening physical harm (Amar & Alexy, 2010; Beatty, 2003; MacKenzie & James, 2011; Weller et al., 2013). In the United States, nationally representative studies indicate that between 1.4 and 5.6 million people are victims of stalking each year, with lifetime victimization estimates between 2% and 8% for men and 7% and 25% for women (Basile et al., 2006; Baum et al., 2009; Black et al., 2011; Catalano, 2012; Tjaden & Thoennes, 1998).

## Stalking Recidivism

Stalking is generally perceived by the public as an intractable problem (Amar & Alexy, 2010; Logan & Walker, 2009; Weller et al., 2013; Yanowitz, 2006), and though not always the case, rates of recidivism do support a need for concern. Follow-up studies examining stalking recidivism have shown that the fraction of offenders who go on to commit a new stalking-related offense is between about one third (e.g., Foellmi et al., 2016; Shea et al., 2018) and one half (e.g., Eke et al., 2011; Mohandie et al., 2006; Rosenfeld, 2003). These studies illustrate the need for effective assessment and subsequent risk management of those that commit stalking offenses.

Nevertheless, our understanding of stalking recidivism remains incomplete, uncertain, and based on a relatively small number of studies. There are two major limitations in the evidence base. First, much of the research has defined stalking recidivism as any new charges or convictions for stalking-related offenses. This definition excludes new stalking that was investigated by police but did not result in charge or conviction or was disposed of by alternative means (e.g., involuntary mental health committal, civil peace bond, or pretrial diversion to a treatment program). Although it is true that some prior studies (e.g., Foellmi et al., 2016; Rosenfeld, 2003) have used other sources of information other than charges and convictions (e.g., subject self-report, information from probation), most stalking recidivism studies have relied on official charges and convictions (i.e., Eke et al., 2011; Malsch et al., 2011; Mohandie et al., 2006; Shea et al., 2018). Official criminal records may not contain sufficient information to determine whether new charges or convictions actually involved stalking-related offenses. For example, without access to additional information, it may be impossible to determine if a new conviction (e.g., for breach of probation) that appears on a criminal record occurred in the context of stalking. Second, much of this research used short- to intermediate-term follow-up periods. Only two studies to date have used an average or median follow-up period of more than 6 years (Eke et al., 2011; Shea et al., 2018). This is problematic because studies have observed significant variability in the duration of stalking offenses (Johnson & Thompson, 2016; McEwan et al., 2017; Purcell et al., 2002). For example, McEwan et al. (2017) found an average duration of about 3 months, but a maximum of about 17.5 years; and Purcell et al. (2002) found an average duration of about 8 months, but a maximum of 40 years. Therefore, short- and intermediate-term follow-up periods may be of insufficient length to capture recidivism among those that engage in persistent stalking.

## Stalking Risk Assessment

Over the past two decades, there has been an effort to develop decision support tools to evaluate stalking risk. They include two sets of structured professional judgment (SPJ) guidelines intended to aid comprehensive, management-oriented risk assessment of stalking perpetrators: the *Guidelines for Stalking Assessment and Management* (SAM; Kropp et al., 2008) and the *Stalking Risk Profile* (SRP; MacKenzie et al., 2009). In this study, we focus on the SAM.

As part of administration of the SAM, evaluators rate the Presence and Relevance of 30 basic factors. The factors were identified from systematic review of the scientific and professional literature as being related to various facets of risk for stalking, including the nature, severity, imminence, frequency, and likelihood of future stalking (Kropp et al., 2008). The factors are presented in Table 1. As the table indicates, the factors are divided into three domains. The Nature of Stalking Factors (N) reflect the pattern, complexity, and severity of the perpetrator’s stalking behavior. The Perpetrator Risk Factors (P) reflect the psychosocial adjustment and background of the perpetrator. The Victim Vulnerability Factors (V) reflect characteristics of the victim’s psychosocial adjustment and living situation that may impair their capacity to engage in self-protective behavior. Based on analysis of the Presence and Relevance ratings for the basic and any case-specific risk factors, evaluators develop individualized formulations of stalking risk and scenario-based case management plans, then make several ratings of overall risk referred to as Conclusory Opinions.

**Table 1.** Domains and Factors in the SAM.

N: Nature of Stalking	P: Perpetrator Risk Factors	V: Victim Vulnerability Factors
N1 Communicates about victim	P1 Angry	V1 Inconsistent behavior toward perpetrator
N2 Communicates with victim	P2 Obsessed	V2 Inconsistent attitude toward perpetrator
N3 Approaches victim	P3 Irrational	V3 Inadequate access to resources
N4 Direct contact with victim	P4 Unrepentant	V4 Unsafe living situation
N5 Intimidates victim	P5 Antisocial lifestyle	V5 Problems caring for dependents
N6 Threatens victim	P6 Intimate relationship problems	V6 Intimate relationship problems
N7 Physically violent toward victim	P7 Non-intimate relationships problems	V7 Non-intimate relationship problems
N8 Stalking is persistent	P8 Distressed	V8 Distressed
N9 Stalking is escalating	P9 Substance use problems	V9 Substance use problems
N10 Stalking involves supervision violations	P10 Employment and financial problems	V10 Employment and financial problems

To date, there have been seven empirical studies utilizing the SAM with stalking offenders (Belfrage & Strand, 2008; Foellmi et al., 2016; Gerbrandij et al., 2018; Kropp et al., 2011; Shea et al., 2018; Storey et al., 2009; Storey & Hart, 2011). With respect to interrater reliability, despite the fact the studies have coded or analyzed risk ratings in different ways, they found Presence and Relevance ratings of the N and P factors typically had moderate to good interrater reliability (ICC_A,1_ = .64–.91), ratings of V factors had fair to good reliability (ICC_A,1_ .= 39–.80), and ratings of Conclusory Opinions had interrater reliability that ranged from fair to good (ICC_A,1_ .= 39–.80). With respect to concurrent validity, the number of factors rated Present in the N, P, and V domains, as well as the Conclusory Opinion ratings, had on average small to medium correlations (*r* = .20–.46) with lifetime symptoms of psychopathy as measured by the Screening Version of the Hare Psychopathy Checklist–Revised or PCL:SV (Hart et al., 1995) and small correlations (*r* = .11–.25) with risk for general violence as measured by the Violence Risk Appraisal Guide or VRAG (Quinsey et al., 1998).

Only two studies to date have examined the predictive validity of the SAM. In the first, Foellmi et al. (2016) assessed 89 offenders referred to a university-based treatment program following conviction for stalking or harassment-related offenses in New York City. The average length of follow-up was 2.51 years (*SD* = 1.38). The authors found that SAM total scores, calculated by summing individual risk factors, provided significant and unique contributions to predicting stalking recidivism, with higher scores associated with more rapid reoffending. When examining the contributions of the N and P factors, it was found that the former significantly contributed in a positive manner to predicting stalking recidivism whereas the latter did not. Conversely, scores on the PCL:SV were negatively associated with stalking recidivism. Additional Cox proportional hazard analyses were conducted which included two separate models using either the N or P factors and PCL:SV total score. A predictive model using the N factors and PCL:SV total score was significant, where higher N factors and lower PCL:SV total scores predicted future stalking. However, SAM Conclusory Opinions were not significantly associated with stalking nor with other (non-stalking) types of violent reoffending, nor were the SAM total scores and domain totals associated with other types of violent reoffending. Foellmi et al. (2016) posited this finding may be due either to limitations of the SAM or, alternatively, methodological limitations of their study—the latter including inability to code V Factors, a low base rate of recidivism involving physical harm (11%) and a very low selection rate of people judged to be at high risk for serious physical harm (1%). In addition, the authors noted that their sample comprised predominantly of low to moderate risk offenders who participated in an intensive treatment program specifically intended to reduce the likelihood of future stalking offenses. Overall, Foellmi et al. (2016) concluded that their study supported the use of the SAM to assess risk for stalking recidivism, and that further research was needed to evaluate whether the SAM had utility in assessing risk for physical harm in the context of stalking.

In the second study, Shea et al. (2018) coded the SAM on a sample of 131 stalking offenders referred to a community-based forensic mental health service in Australia. This study examined whether recidivism was targeted against the same person victimized in the index offense or against another person. The median length of time for follow-up was approximately 6 years, and recidivism was coded from official police records and included criminal charges and formal interventions (e.g., restraining orders). Stalking recidivism, defined by the presence of a new stalking charge or evidence of a pattern of charges against the same victim, was present in 17% of the sample, whereas recidivism against a new victim was present in 13% of the sample. Case Prioritization ratings showed the sample to be predominantly low and moderate risk. Using Kaplan–Meier survival analysis, individuals rated as low on Case Prioritization and Risk for Continued Stalking were found to reoffend against the same victim significantly less often and more slowly than those judged as moderate or high risk. Specifically, those rated as high risk were five to nine times more likely to reoffend against their index victims. Limitations of the study included the inability to examine the V Factors, low base rates of physical harm (5.3%) that prevented predictive validity analyses on this outcome, and a definition of recidivism that required the presence of legal charges. Shea et al. (2018) concluded that their study supported the use of the SAM when assessing risk for future stalking.

This current study sought to build on Foellmi et al. (2016) and Shea et al. (2018), while addressing some of the limitations of previous research in understanding stalking recidivism. Specifically, the objectives of the current study were (a) to characterize the long-term risk for recidivism in a sample of offenders convicted of stalking-related offenses in Canada and (b) to evaluate the predictive validity of judgments of risk for stalking made using the SAM with respect to recidivism, including their incremental predictive validity with respect to total scores on the PCL:SV and VRAG. It was anticipated that those individuals who had more SAM risk factors rated as present and higher Conclusory Opinion ratings would be more likely to reoffend, consistent with the existing research on the SAM. It was also hypothesized that the SAM would contribute incremental predictive validity beyond the PCL:SV and VRAG.

## Method

### Overview

We report how we determined our sample size, all data exclusions, all manipulations, and all measures in the study. We identified a cohort of 106 adult offenders who were adjudicated (found guilty or Not Criminally Responsible on Account of Mental Disorder) for stalking-related offenses under the *Criminal Code* of Canada (R. S. C. 1985, c. C-46) and who underwent post-adjudication assessment (e.g., to evaluate violence risk or to provide diagnostic clarification) or treatment at a community outpatient clinic of the British Columbia Forensic Psychiatric Services Commission (FPSC) and were discharged between April 1, 1992 and March 31, 2009. Stalking-related offenses included criminal harassment, trespass, loitering, uttering threats, assaults, harassing phone calls, and intimidation that occurred in the context of a pattern of conduct that recklessly or deliberately caused victims to fear for their own safety or the safety of others known to them. For the purpose of the current study, we excluded two offenders from the cohort for whom we could not collect follow-up data and four additional offenders who were missing data on one of the risk measures analyzed, yielding a final sample of 100 offenders. The current sample overlaps partially with those of two published studies of stalking-related offenses and offenders (Kropp et al., 2011; Storey et al., 2009), but neither of those prior studies examined stalking recidivism or the predictive validity of ratings made using the SAM.

The current study received approval from Office of Research Ethics at Simon Fraser University, the FPSC, and the Vancouver Police Department.

### Sample Characteristics

With respect to demographic characteristics, the mean age of the offenders at the time of discharge from the FPSC clinic was 36.89 years (*SD* = 10.40, range = 19–73). Most offenders were male (89%) and of European descent (75%). A substantial minority of offenders (26%) had a history of being diagnosed with symptoms of psychotic disorders, including substance-related psychotic disorders.

With respect to offense characteristics, the primary victim was a lone female in 78% of cases and a lone male in 19% of cases; in the remaining cases, there were multiple primary victims or a corporate victim (i.e., a governmental, nongovernmental, or private organization). The offender and victim(s) were family members or former intimate partners in 53% of cases, acquaintances in 30% of cases, and strangers in 17% of cases.

### Procedure

The study utilized a retrospective follow-up or quasi-prospective design. Risk measures were coded retrospectively from FPSC files by researchers, all of whom were senior graduate students in forensic psychology with extensive training or experience in the use of the risk measures. The files were coded between April 1, 2009 and December 31, 2011 on the basis of information about offenders gathered in the course of their assessment or treatment up to that point in time (i.e., at discharge). For each case, either two or three researchers coded files blind to the ratings made by each other and, importantly, blind to outcome. Researchers then reviewed the independent ratings for each case and made a final set of consensus ratings that were intended to maximize the validity of the risk ratings. The consensus procedure increases the accuracy of ratings, and consequently, statistical power for analyses based on them. The independent ratings were used in analyses of interrater reliability, and the consensus ratings were used in analyses of concurrent and predictive validity.

Recidivism data were collected and coded in 2013 by an analyst in the Vancouver Police Department’s Domestic Violence and Criminal Harassment (DVACH) unit. For all offenders, the analyst reviewed multiple electronic police and criminal record databases and coded all their contacts with police after they were discharged from the FPSC clinic.

### Materials

#### SAM

Administration of the SAM involves six steps (Kropp et al., 2008). In Step 1, evaluators gather and review case information. In Step 2, evaluators code the presence of the 30 basic risk factors, plus any case-specific risk factors, across two timeframes: Recent, during the current pattern of stalking behavior; and Past, prior to the current pattern. Presence ratings reflect evidence that a risk factor is observed in the case and are made on a 3-point ordinal scale (*Yes*, *Possibly or Partially*, *No*). In Step 3, evaluators develop a narrative case formulation of the perpetrator’s stalking behavior. They identify the P and V factors that appeared to have played important causal roles in the person’s decisions to engage in stalking behavior and may be relevant to the perpetration or prevention of future stalking, and code the relevance of those factors on a 3-point ordinal scale (*Yes*, *Possibly or Partially*, *No*). In Step 4, evaluators develop narratives of what they considered to be the primary plausible scenarios of future stalking. For each plausible scenario, they describe the nature, seriousness, imminence, frequency or duration, and likelihood of potential future stalking. In Step 5, evaluators recommend management plans in light of the scenarios of stalking violence they had identified. The recommendations were divided into four major categories: monitoring, treatment, supervision, and victim safety planning. In Step 6, evaluators form Conclusory Opinions regarding several specific aspects of overall risk for future stalking, including Case Prioritization (level of effort or intervention required to prevent future stalking), Continued Stalking (risk that stalking will persist in any form), Serious Physical Harm (risk that stalking may involve lethal or life-threatening violence severity), Reasonableness of Fear (the degree to which the victim’s concern for their own safety appears to be appropriate, that is, neither too low nor to high, in light of the evaluator’s opinions concerning risk), and Immediate Action Required (risk that stalking may occur in the near future). Conclusory Opinions are expressed using a 3-point ordinal scale (*High*, *Moderate*, and *Low*).

The SAM was administered on the basis of a comprehensive file review. Material contained within the files typically included but was not limited to: police reports including a narrative of the index offense, previous criminal record, victim interviews, and available medical records. In cases where there were multiple primary victims, one set of Victim Vulnerability factors was coded based on the risk factors present across the victims. Researchers relied on the draft version of the SAM manual available at the time data were collected, which differed from the final version in that Relevance ratings were not yet included.

For the purpose of the present study, we focused on the predictive validity of consensus ratings of Presence and the three major Conclusory Opinions (Case Prioritization, Continued Stalking, and Serious Physical Harm). We estimated the interrater reliability of the consensus ratings from that of ratings made by independent researchers. There has been considerable debate regarding how best to index interrater reliability (e.g., Cicchetti et al., 2017; Zhao et al., 2013), and each approach has limitations (Feng, 2013). To avoid issues related to the so-called kappa paradox (i.e., the artificial reduction of interrater reliability coefficients resulting from limited variance in samples; see Feinstein & Cichetti, 1990; Gwet, 2014), we elected to use Gwet’s AC agreement statistic. We interpreted AC values using the Landis and Koch (1977) guidelines.

Table OSM1 of the online supplemental materials presents the distribution for consensus Presence ratings, Recent and Past, for individual SAM factors, as well as their interrater reliability. With respect to interrater reliability, the AC_2_ values ranged from a minimum of .56 to a maximum of .95, with a *Mdn* of .82; most (55%) fell in the category labeled *almost perfect* by Landis and Koch (1977). The distribution and interrater reliability of consensus ratings for the Conclusory Opinions was as follows: Case Prioritization, *Low* = 9%, *Moderate* = 56%, *High* = 35%, AC_2_ = .76, 95% confidence interval (CI) = [.68 .85]; Continued Stalking, *Low* = 12%, *Moderate* = 44%, *High* = 44%, AC_2_ = .76, 95% CI = [.59, .92]; and Serious Physical Harm, *Low* = 45%, *Moderate* = 42%, *High* = 12%, AC_2_ = .71, 95% CI = [.55, .87]. The interrater reliability for all three Conclusory Opinions fell in the category labeled *substantial* by Landis and Koch (1977).

#### Recoding Predictors for Analysis

Consistent with past research on SPJ guidelines (see Douglas & Otto, 2021), we converted the SAM ratings for Presence and Conclusory Opinions into numeric scores for analyses of concurrent and predictive validity. To reduce the number of analyses, we recoded the Past and Recent ratings for each factor (3 = *yes*, 2 = *possibly or partially*, 1 = *no*) and multiplied them to create a single Ever Present score (1 = *low*, 9 = *high*). We elected to multiply rather than sum the Presence ratings, as multiplication produced a more normal distribution of values and is more punitive for those who have engaged in persistent stalking. We then summed the Ever Present scores for individual factors to yield a total score. (Note that this Ever Present composite is a formative index intended to evaluate the predictive validity of SAM factors as an ensemble; because the SAM factors were identified solely on the basis of their association with stalking risk, there is no reason to assume the individual Presence ratings or Ever Present composite scores reflect one or more latent variables.) For Conclusory Opinions, we recoded ratings into numerical scores (0 = *low*, 1 = *moderate*, 2 = *high*).

#### PCL:SV

The PCL:SV (Hart et al., 1995) is a 12-item symptom construct rating scale of the lifetime presence of features of psychopathic personality disorder, derived from the 20-item Hare Psychopathy Checklist–Revised (PCL-R; Hare, 2003). The psychometric properties of the PCL:SV have been well established within the context of modern test theory (for a brief review, see Hart & Wilson, 2008). Each of the 12 items in the PCL:SV is coded on a 3-point ordinal scale (briefly, 0 = *item does not apply*, 1 = *item applies to some extent*, and 2 = *item applies*). The 12 items may be summed to yield total scores ranging from 0 to 24.

In the present study, the PCL:SV was scored according to the instructions in Hart et al. (1995) based on comprehensive file information. PCL:SV ratings could not be made for one participant due to missing information. As noted previously, two researchers made independent PCL:SV ratings for each case, after which the blind was broken and the independent ratings were combined to make a single set of consensus ratings. The consensus PCL:SV total scores ranged from 1 to 20, *M* = 9.02 and *SD* = 4.35. The interrater reliability of PCL:SV total scores was AC_2_ = .73, 95% CI = [.54, .92], which falls in the category labeled *substantial agreement* by Landis and Koch (1977).

#### VRAG

The VRAG (Quinsey et al., 1998) is an actuarial scale developed to assess risk for violent recidivism. It comprises 12 items that are summed to yield total scores ranging from −25 to 38. The total scores are then divided into nine “bins,” each of which is associated with a likelihood of violent reoffending over 7- and 10-year follow-up periods. As summarized by the authors, research on VRAG ratings has reported high interrater reliability as well as moderate to high predictive validity for general and violent recidivism (e.g., Harris et al., 2015).

In the present study, the VRAG was scored according to the instructions in Quinsey et al. (1998) on the basis of comprehensive file information, with one exception: Item 1, which reflects scores on the PCL-R, was calculated digitally by taking the consensus PCL:SV rating for each participant and converting it to a total score on the PCL-R using information in Cooke et al. (1999). As noted previously, two researchers made independent VRAG ratings for each case, after which the blind was broken and the independent ratings were combined by the researchers to make a single set of consensus ratings. The consensus VRAG total scores ranged from −18 to 19, *M* = −3.03 and *SD* = 8.11. The interrater reliability of VRAG total scores was AC_2_ = .91, 95% CI = [.81, 1.00], which falls in the category labeled *almost perfect agreement* by Landis and Koch (1977).

#### Recidivism

Recidivism was coded from official police records and involved a comprehensive search of federal, provincial, and municipal databases, including the Canadian Police Information Centre, Criminal Names Index, and Police Records Information Management Environment (and its predecessor, the Police Information Retrieval System). The analyst reviewed all police incident reports reflecting contacts with each offender after the date of their discharge from the FPSC clinic, working blind to information regarding the index offense, the content of the FPSC clinic files, and risk measure ratings made by the researchers. For each contact, the analyst coded (a) the date of the contact, (b) whether the participant was identified as the suspect of one or more alleged offenses, (c) the nature of any alleged offenses, and (d) whether the individual was charged or convicted in relation to any alleged offenses. Criminal record searches were conducted in January 2013. Recidivism was defined as any police contact wherein the individual was identified as the suspect of an alleged offense. Stalking recidivism was defined as consistent with the coding of index offenses (i.e., behavior that included: criminal harassment, trespass, loitering, uttering threats, assaults, harassing phone calls, and intimidation that occurred in the context of a pattern of conduct that recklessly or deliberately caused victims to fear for their own safety or the safety of others known to them). Patterns of conduct were identified on the basis that (a) the alleged perpetrator was the same in each incident, (b) the victim was the same in each incident, (c) two or more instances of fear-inducing behavior were described even if only one report to police was made (e.g., if a victim described behavior occurring over multiple days but included all instances in one report to police). Violent recidivism (which included stalking offenses) was defined as any actual, attempted or threatened harm to a person or persons. Due to privacy concerns, it was not possible to collect identifiable victim information, which would have indicated whether the victim was the same victim as the index offense or whether recidivism occurred against a new victim.

For the purpose of coding data, the end of the follow-up period was set at January 1, 2013, with the exception that date of death was set as the end of the follow-up period for three individuals who died prior to January 1, 2013. The possible follow-up time for offenders, calculated as time between date of discharge and date of the end of follow-up, ranged from 382 days to 7,564 days (*Mdn* = 4,920 days, interquartile range [IQR] = 3,192.5–6,118.5 days). These figures correspond to a maximum possible follow-up time of 20.71 years and a median possible follow-up time of 13.47 years.

We coded recidivism (failure) and survival time (time to failure) in a number of ways, but for the purpose of this study, we focus on any stalking, defined as any new conviction, charge, or police investigation for any stalking-related offenses after discharge. Survival time for recidivists was defined as the number of days between (a) discharge from the FPSC clinic and (b) date of first contact with police in relation to the conviction, charge, or investigation for any stalking. Survival time for those coded as non-recidivists was defined as the number of days between discharge from the FPSC clinic and (b) the date of death or January 1, 2013, whichever came first.

### Data Analyses

All data analyses were conducted using Stata/SE, version 14.2.

Survival analyses comparing the predictive validity of the SAM, PCL:SV, and VRAG were complicated by the fact that it is impossible to directly compare effect sizes for predictors with different metrics. (The magnitude of the hazard ratio [HR] for a predictor reflects the increase in relative risk associated with a 1-unit increase in the value of that predictor, so the magnitude of the HRs are directly comparable only when the predictor variables have the same metric.) To facilitate comparison, we used Stata to automatically recode SAM Ever Present, PCL:SV total, and VRAG total scores into quintiles, that is, five groups of approximately equal size (1 = *low*, 5 = *high*). The Pearson product–moment correlations among the SAM Ever Present, PCL:SV, and VRAG quintile scores, as well as their correlations with SAM Conclusory Opinion ratings, are presented in Table 2.

**Table 2.** Correlations Among SAM Ever Present and Conclusory Opinion Scores and PCL-R and VRAG Total Scores.

Variable	1	2	3	4	5	6
1. SAM total (quintiles)		.076	.046	**.248**	**.431**	**.272**
2. SAM case prioritization	.451		.**574**	.**349**	**.318**	**.258**
3. SAM continued stalking	.648	<.001		**.292**	**.203**	.085
4. SAM serious physical harm	.014	<.001	.003		.186	.179
5. PCL:SV total (quintiles)	<.001	.001	.043	.066		.**564**
6. VRAG total (quintiles)	.006	.010	.404	.076	<.001	

*Note. N* = 100. SAM = Guidelines for Stalking Assessment and Management; PCL:SV = Screening Version of the Hare Psychopathy Checklist–Revised; VRAG = Violence Risk Appraisal Guide. Correlations (*r*) are presented above the diagonal, and corresponding significance (*p*) levels are presented below the diagonal. Correlations significant at *p* < .05 are highlighted in bold.

## Results

### Any Stalking Recidivism

The observed recidivism rate (proportion) for any stalking at the end of the follow-up period was .45, 95% CI = [.35, .55]. Using broader definitions of recidivism (which included any stalking), the observed rate for any violence was .57, 95% CI = [.47, .66], and the observed rate for any offense was .76, 95% CI = [.67, .83]. These figures indicate that most of those who recidivated in the sample did so by engaging in stalking-related offenses.

Focusing on the 45 recidivists who engaged in any stalking, 6 were convicted of a new stalking-related offense, 18 were charged with a new stalking-related offense, and 21 had police contact for investigation of a new stalking-related offense. Only two instances of any stalking recidivism involved actual or attempted serious physical harm to victims, yielding a rate of .02, 95% CI = [.00, .07]; one instance resulted in a charge for a new stalking-related offense, and the other resulted in police contact for investigation of a new stalking-related offense.

**Figure 1.**
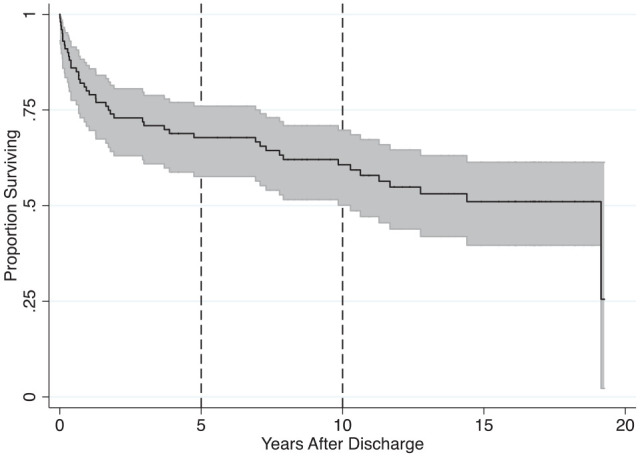
Kaplan–Meier Survival Function: Any Stalking-Related Incidents After Discharge (Reference Lines Added at 5 and 10 Years).

The observed recidivism rates are a lower-bound estimate, as they are not adjusted for between-subject heterogeneity in time at risk (also known as right-hand censoring) due to differences in date of discharge; convictions, charges, or police contact for other violent or nonviolent offenses; and death. Furthermore, the observed rates do not provide information concerning the rates of recidivism as a function of time (also known as hazard rates). We characterized any stalking recidivism as a function of time after discharge using survival analysis. The Kaplan–Meier survivor function for any stalking recidivism is presented in Figure 1. As is evident in the figure, the first instance of any stalking recidivism occurred rapidly, only 1 day after discharge, and the last occurred near the end of the follow-up, more than 19 years after discharge. The Kaplan–Meier failure function (the complement of the survival function), which is an estimate of the hazard rate after adjustment for right-hand censoring, at 5 years after discharge was .32, 95% CI = [.24, .42]; at 10 years after discharge, .39, 95% CI = [.30, .50]; and at 20 years after discharge, .74, 95% CI = [.39, .98].

### Predictive Validity of the SAM

#### Modeling Recidivism

We conducted survival analyses using the Cox proportional hazards model to analyze predictive validity. The Cox model permits univariate and multivariate analysis of predictors to determine their impact on the hazard rate. Below, we evaluated overall model fit using a chi-square test and the validity of individual predictors in the model in terms of their associated HRs.

#### Impact of Demographic and Offense Characteristics

Prior to undertaking substantive analyses, we examined the extent to which the demographic characteristics (age at discharge, gender, ethnicity, history of psychosis) and offense characteristics (gender of victim, acquaintanceship with victim) were related to recidivism. None of these variables was significantly associated with recidivism, either individually or jointly. We, therefore, did not include them as control or nuisance variables in subsequent analyses.

#### SAM Conclusory Opinions

We began by evaluating the predictive validity of SAM Conclusory Opinions with respect to time to recidivism at 5, 10, and 20 years after discharge. We tested the predictive validity of the three SAM Conclusory Opinion ratings jointly. The results are also presented in Table 5. We evaluated potential problems due to violation of the proportional hazards assumption, but the slopes of the regression of scaled Schoenfeld residuals on time were not significantly different from 0 in any analysis, all *p* >> .05. We also evaluated potential problems due to collinearity among predictors, as indicated by a variance inflation factor (VIF) of 4 or greater, or other problems, such as “failure to converge” errors, extremely large HRs, or HRs with missing SE estimates; there were none.

At all three time gates, there was no indication that the ratings were significantly associated with any stalking recidivism, either individually or jointly. Looking at the individual ratings, Risk for Continued Stalking had a nonsignificant positive association with recidivism at all three time gates and Case Prioritization had a nonsignificant negative association with recidivism at all three time gates; Risk for Physical Harm had a very small association with recidivism at all three time gates, which was varied in direction.

#### Individual SAM Factors

To evaluate the predictive validity of individual SAM factors with respect to time to recidivism at 5, 10, and 20 years after discharge, we conducted 90 univariate analyses, one for each of 30 factors at the three different time gates. The findings are summarized in Table 3. We evaluated potential problems due to violation of the proportional hazards assumption (i.e., that HRs are constant over time), as indicated non-zero slope in a generalized linear regression of the scaled Schoenfeld residuals on time, *p* < .05. As indicated in Table 3, these were observed in seven analyses, which is close to the number that would be expected on the basis of chance (i.e., 90 × .05 ≈ 5).

**Table 3.** Cox Regression Survival Analyses for Prediction of Any Stalking at 5, 10, and 20 Years After Discharge: Ever Present Ratings of Individual SAM Factors.

	5 Years		10 Years		20 Years	
SAM domain/factor	HR	*p*	HR	*p*	HR	*p*
**Nature of Stalking**						
N1 Communicates about victim	**1.16** *	.017	**1.16**	.015	**1.16**	.016
N2 Communicates with victim	**1.17** *	.005	**1.17**	.003	**1.17**	.002
N3 Approaches victim	.98	.076	.97	.680	.96	.530
N4 Direct contact with victim	1.08	.271	1.04	.527	1.01	.931
N5 Intimidates victim	**1.16** *	.012	**1.15**	.011	**1.14** *	.011
N6 Threatens victim	**1.23** *	.001	**1.20**	.002	**1.18**	.004
N7 Physically violent toward victim	1.14	.153	1.09	.345	1.10	.276
N8 Stalking is persistent	**1.18**	.010	**1.14**	.032	**1.13**	.044
N9 Stalking is escalating	1.09*	.239	1.06	.377	1.06	.391
N10 Stalking involves supervision violations	**1.20**	.001	**1.18**	.001	**1.19**	<.001
**Perpetrator Risk Factors**						
P1 Angry	**1.25**	.001	**1.19** *	.002	**1.14**	.007
P2 Obsessed	**1.13**	.028	**1.12**	.035	1.09	.099
P3 Irrational	1.09	.121	1.07	.204	1.06	.265
P4 Unrepentant	**1.19**	.003	**1.14**	.012	**1.12**	.017
P5 Antisocial lifestyle	1.08	.164	1.06	.274	1.08	.077
P6 Intimate relationship problems	**1.17**	.009	**1.12**	.029	1.08	.092
P7 Non-intimate relationships problems	1.08	.195	1.03	.354	1.01	.812
P8 Distressed	1.02	.753	1.02	.735	1.00	.948
P9 Substance use problems	1.01	.888	1.01	.994	.99	.910
P10 Employment and financial problems	1.12	.051	**1.14**	.016	**1.11**	.041
**Victim Vulnerability Factors**						
V1 Inconsistent behavior toward perpetrator	**1.15**	.009	**1.12**	.046	1.09	.110
V2 Inconsistent attitude toward perpetrator	1.14	.067	1.10	.190	1.08	.273
V3 Inadequate access to resources	.71	.359	.85	.539	.79	.391
V4 Unsafe living situation	**1.15**	.018	1.12	.053	1.09	.115
V5 Problems caring for dependents	1.12	.052	1.10	.106	1.08	.186
V6 Intimate relationship problems	**1.14**	.011	**1.11**	.033	1.08	.115
V7 Non-intimate relationship problems	**1.19**	.047	1.15	.099	1.12	.224
V8 Distressed	**1.34**	<.001	**1.32**	<.001	**1.29**	<.001
V9 Substance use problems	.31	.193	.25	.135	.37	.116
V10 Employment and financial problems	1.04	.867	.94	.804	.83*	.460

*Note. N* = 100. SAM = Guidelines for Stalking Assessment and Management; HR = hazard ratio. HRs significant at *p* < .05 are highlighted in bold. HRs marked with an asterisk (*) indicate a potential violation of the proportional hazards assumption as indicated by non-zero slope in a generalized linear regression of the scaled Schoenfeld residuals on time, *p* < .05.

Looking first at the N domain, 6 of 10 factors were positively and significantly related to recidivism at all three time gates: N1 (Communicates about victim), N2 (Communicates with victim), N5 (Intimidates victim), N6 (Threatens victim), N7 (Stalking is persistent), and N10 (Stalking involves supervision violations). Of the other factors, three were positively but nonsignificantly related to recidivism at all three time gates and 1 was negatively but non-significantly related to recidivism at all three time gates.

Next, turning to the P domain, 2 of the 10 factors were positively and significantly related to recidivism at all three time gates: P1 (Angry) and P4 (Unrepentant). Another three factors were positively and significantly related to recidivism at two of three time gates: P2 (Obsessed), P6 (Intimate relationship problems), and P10 (Employment and financial problems). Of the other 5 factors, 4 were positively but non-significantly related to recidivism at all three time gates and 1 was positively but non-significantly related to recidivism at a single time gate.

Finally, looking at the V domain, one factor was positively and significantly related to recidivism at all three time gates: V8 (Distressed). Another 2 factors were positively and significantly related to recidivism at two time gate: V1 (Inconsistent behavior toward perpetrator) and V6 (Intimate relationship problems). Another two factors were positively and significantly related to recidivism at a single time gate: V4 (Unsafe living situation) and V7 (Non-intimate relationship problems). Of the other factors, two were positively but nonsignificantly related to recidivism at all times gates and one was positively but nonsignificantly related to recidivism at a single time gate; two were negatively but nonsignificantly related to recidivism at all time gates.

Overall, then, the findings provided support for the predictive validity of 16 SAM factors: 9 had a positive and significant association with recidivism at all three time gates; 5 factors had a positive and significant association with recidivism at two time gates; and 2 factors had a positive and significant association with recidivism at one time gate. In addition, most of the remaining factors had some positive associations with recidivism: seven had associations that were positive but not statistically significant at all three time gates; two had associations that were positive but not statistically significant at two time gates, and two had associations that were positive but not statistically significant at one time gate. Only 3 of 30 factors (10%) had negative associations with recidivism at all three time gates, and none of them was statistically significant.

#### SAM Ever Present Composite

As noted previously, we constructed an Ever Present composite to test the predictive performance of the SAM factors as an ensemble. If the individual SAM factors were, on average, valid predictors of recidivism, as the findings of the univariate analyses indicated, then the composite may also be a valid predictor of recidivism. (An alternative test of the factors as an ensemble is to enter each of them, and even interactions among them, in a single multivariate analysis, but such an approach would require a much larger sample.) The predictive validity of the SAM Ever Present composite at 5, 10, and 20 years after discharge is presented in Table 4. We evaluated potential problems due to violation of the proportional hazards assumption, but the slope of the regression of scaled Schoenfeld residuals on time was not significant in any analysis, all *p* >> .05.

**Table 4.** Cox Regression Survival Analyses for Prediction of Any Stalking at 5, 10, and 20 Years After Discharge: SAM Ever Present and Conclusory Opinions.

	5 Years			10 Years			20 Years		
Model/predictor	HR	95% CI	*p*	HR	95% CI	*p*	HR	95% CI	*p*
**Ever present**									
Total (quintiles)	**1.73**	[1.30, 2.29]	<.001	**1.51**	[1.19, 1.93]	.001	**1.36**	[1.09, 1.70]	.007
Model fit	χ^2^(1) *=* 16.53, *p* < .001			χ^2^(1) *=* 11.87, *p* < .001			χ^2^(1) *=* 7.57, *p* = .006		
**Conclusory opinions**									
Case prioritization	.88	[.44, 1.77]	.736	.93	[.49, 1.77]	.820	.90	[.49, 1.65]	.736
Continued stalking	1.69	[.88, 3.26]	.191	1.58	[.87, 2.89]	.135	1.46	[.83, 2.56]	.191
Physical harm	1.05	[.62, 1.79]	.952	.96	[.59, 1.58]	.881	.99	[.63, 1.55]	.952
Model fit	χ^2^(3) *=* 3.19, *p* = .363			χ^2^(3) *=* 2.82, *p* = .420			χ^2^(3) *=* 2.04, *p* = .564		

*Note. N* = 100. HR = hazard ratio; SAM = Guidelines for Stalking Assessment and Management. HRs significant at *p* < .05 are highlighted in bold.

At all three time gates, the SAM Ever Present composite was positively and significantly associated with any stalking recidivism. The HRs indicated that each one-step increase in the composite score (which, recall, had been recoded into quintiles) was associated with an increase in the risk hazard rate of recidivism of between 36% and 73%.

#### Incremental Validity of SAM Ever Present Composite

Finally, we tested the extent to which the SAM Ever Present composite predicted any stalking recidivism relative to total scores on two risk-relevant measures used in prior research, the PCL:SV and VRAG. We did this by comparing the fit of two predictive models: the first included PCL:SV and VRAG scores, and the second included PCL:SV, VRAG, and SAM Ever Present scores. The results are presented in Table 5. In all the analyses, there was no indication of violation of the proportional hazards assumption (slopes of the regression of scaled Schoenfeld residuals on time were not significantly greater than 0, all *p* >> .05), problems due to collinearity among predictors (all VIFs < 4), or problems due to “failure to converge” errors, extremely large HRs, or HRs with missing *SE* estimates.

**Table 5.** Cox Regression Survival Analyses for Prediction of Any Stalking at 5, 10, and 20 Years After Discharge: Incremental Validity of SAM Ever Present Versus PCL-R and VRAG.

	5 Years			10 Years			20 Years		
Step/predictor	HR	95% CI	*p*	HR	95% CI	*p*	HR	95% CI	*p*
**Step 1**									
PCL: SV total (quintiles)	1.32	[.99, 1.77]	.058	**1.33**	[1.03, 1.73]	.033	1.17	[.93, 1.49]	.188
VRAG total (quintiles)	0.95	[.71, 1.28]	.753	0.91	[.70, 1.19]	.482	1.01	[.79, 1.30]	.906
Model fit	χ^2^(2) *=* 4.31, *p* = .116			χ^2^(2) *=* 4.84, *p* = .089			χ^2^(2) *=* 2.54, *p* = .281		
**Step 2**									
PCL:SV total (quintiles)	1.14	[.83, 1.58]	.416	1.20	[.91, 1.60]	.198	1.08	[.84, 1.39]	.550
VRAG total (quintiles)	0.89	[.65, 1.20]	.441	0.84	[.64, 1.11]	.230	0.97	[.75, 1.25]	.793
Ever present total (quintiles)	**1.71**	[1.26, 2.31]	<.001	**1.49**	[1.15, 1.95]	.003	**1.33**	[1.05, 1.70]	.020
Change in model fit	χ^2^_*diff*_(1) = 13.03, *p* = .002			χ^2^_*diff*_(1) *=* 9.05, *p* = .003			χ^2^_*diff*_(1) *=* .39, *p* = .020		

*Note. N* = 100. HR = hazard ratio; SAM = Guidelines for Stalking Assessment and Management; PCL:SV = Screening Version of the Hare Psychopathy Checklist–Revised; VRAG = Violence Risk Appraisal Guide. HRs significant at *p* < .05 are highlighted in bold.

The first model was not significantly associated with recidivism at any of the three time gates. Looking at the predictors individually, PCL:SV total scores were positively associated with recidivism at all three time gates, and at one time gate, the association was statistically significant. In contrast, VRAG total scores had a very small and nonsignificant association with recidivism at all three time gates that was varied in direction. Adding SAM Ever Present composite scores in the second model led to a significant improvement in model fit at all three time gates. Looking at the predictors individually, SAM Ever Present composites had a positive and statistically significant association with recidivism at all three times gates. In contrast, PCL:SV total scores were positively but not significantly associated with recidivism at all three time gates and VRAG total scores were negatively but not significantly associated with recidivism at all three time gates.

## Discussion

The first objective of our study was to characterize the long-term risk for stalking recidivism in a sample of offenders from Canada. We were able to gather a moderate-sized sample of 100 offenders adjudicated for stalking-related offenses who were seen at an outpatient forensic clinic and follow them up via a comprehensive search of police databases for an average of 13.47 years and a maximum of 20.71 years. The results revealed that 45 offenders had convictions, charges, or police contacts for investigation of any stalking-related offenses during the follow-up period. Another 31 offenders had convictions, charges, or police contacts for investigation of offenses that were not confirmed to be stalking-related; in 12 cases, at least one of the offenses was violent in nature and in the remaining 19 cases all the offenses were nonviolent in nature. Adjustment for between-subject heterogeneity in time at risk revealed that the estimated stalking recidivism was 32% at 5 years after discharge from the clinic, 39% at 10 years after discharge, and 74% at 20 years after discharge. These findings are consistent with the nascent literature on stalking recidivism that was summarized in the Introduction. They are notable for being based on the longest follow-up period to date in the research literature, and also the first comprehensive description of stalking recidivism in a sample of Canadian offenders.

The second objective of the current study was to evaluate the usefulness of the SAM with respect to assessing risk for stalking recidivism. We tested the predictive validity of Ever Present ratings for SAM factors. The findings provided support for the predictive validity of the SAM factors individually, as just over half of them were positively and significantly associated with stalking recidivism at one or more time gates and none of them was negatively and significantly associated with stalking recidivism at any time gate. The findings also supported the predictive validity of the SAM factors as an ensemble as indexed by a composite total score, which was positively and significantly associated with stalking recidivism at all three time gates. This composite also had positive and significant incremental predictive validity compared with total scores on the PCL:SV and VRAG. Furthermore, we tested the predictive validity of Conclusory Opinion ratings made using the SAM. These were not positively and significantly associated with recidivism at any time gate; this rendered moot the need to test their incremental predictive validity compared with PCL:SV and VRAG total scores.

These findings are generally consistent with the literature supporting the predictive validity of the SAM, with the exception of the findings regarding the Conclusory Opinion ratings made using the SAM, which have previously been found to be associated with recidivism (Foellmi et al., 2016; Shea et al., 2018). It is possible that the discrepancy in this study’s findings may be related to the nature of the current sample, such that it was a moderate risk sample with dynamic risk factors which were being managed reasonably well by multidisciplinary forensic mental health teams to reduce the likelihood of further involvement with the criminal justice system. This may have led to decreased predictive validity as interventions may have been effective at mitigating reoffending. Consistent with this explanation, when indexing stalking recidivism using formal charges and convictions, only one quarter of the sample reoffended, which represents a lower recidivism rate than previous research findings (Eke et al., 2011; Foellmi et al., 2016; Mohandie et al., 2006; Rosenfeld, 2003; Shea et al., 2018). This study’s findings are also consistent with the more general literature supporting the interrater reliability and predictive validity of violence risk judgments made using SPJ guidelines and other structured decision support aids (for reviews, see Douglas & Otto, 2021).

### Implications for Future Research

The current findings provided additional evidence supporting the predictive validity, interrater reliability, and concurrent validity of risk judgments made using the SAM. But any conclusions must be tempered in light of limitations of our study design. Below, we discuss the major limitations and their implications for future research.

#### Sample

The statistical power of analyses was limited both by the moderate size of the sample size and, consequently, by the heterogeneity of offenders which could not be properly controlled or analyzed. Also, this study was based on offenders attending a forensic mental health outpatient clinic. As a result, it was likely biased by an over-representation of moderate seriousness offenders with mental health problems, as low seriousness offenders or those without mental health problems were likely sentenced to regular community supervision (without assessment or treatment) and high seriousness offenders were likely sentenced to long-term imprisonment. Future research should attempt to study large samples or samples from diverse settings.

Furthermore, as it was not possible to determine whether any victims of recidivistic stalking were the original victims, the relevance of victim-specific factors is also unknown, which appears to be an on-going challenge in the evaluation of the SAM. Future research should endeavor to examine the utility and validity of these ratings in the assessment of stalking risk.

#### SAM Assessments

We were able to make file-based SAM ratings for only a single point in time, namely, at discharge from the forensic mental health outpatient clinic. We then relied on these ratings to model recidivism over a follow-up that averaged more than 13 years and ranged up to almost 21 years. But violence risk is dynamic: both the presence and relevance of risk factors, as well as various facets of overall risk, is expected to fluctuate over time. Reliance on a single assessment tends to underestimate predictive validity. Future research should examine the predictive validity of the SAM using a longitudinal design in which assessments are conducted at multiple timepoints. This would permit analysis of whether and how ratings of risk and the likelihood of actual recidivism may change during the follow-up. Several recent studies have used such designs using other SPJ guidelines (e.g., Hogan & Olver, 2019; Michel et al., 2013; Wilson et al., 2013). Ideally, future research should also expand the assessments to include not only file review but also interviews with both perpetrators and victims, and to focus not only on risk factors but also any other life circumstances that may have affected risk, including such things as case management interventions, physical health problems, and institutionalization (e.g., incarceration or involuntary hospitalization).

#### Multiple Raters

We analyzed consensus ratings made by independent evaluators. This is rare, but not unheard of, in field settings; it is more common to have multiple evaluators work together as a team. Future research should examine SAM ratings made by multiple (three or more) raters across multiple time points, working either independently or as a team. Ideally, reliability across ratings, raters, and time could be evaluated using a Generalizability (G) or Decision (D) Theory (e.g., see Sea & Bang, 2021; Sea & Hart, 2021).

#### Statistical Approaches

To evaluate the predictive validity of the SAM, qualitative descriptors were quantified (e.g., through the calculation of the SAM Ever Present composite score), which differs from the real-world use of the SAM. It is possible that the specific approaches chosen to achieve this may have led to decreased external validity or limitations on generalizability. Furthermore, the decision to transform SAM Ever Present, PCL:SV, and VRAG scores into quintiles to facilitate the comparison between the predictors in the predictive validity analyses may decrease the generalizability of the current findings as the quintiles were generated based on the actual scores obtained from the current sample. Future studies should continue to examine the predictive validity of the SAM using multi-method statistical approaches to better address this limitation.

#### Definition of Recidivism

We included contacts with police in our definition of recidivism, whereas most stalking research has defined recidivism in terms of arrest, charge, or conviction. There is, of course, no single correct definition of recidivism. Definitions that rely on official record data (e.g., criminal records) will inherently underestimate the “true” recidivism rate due to reliance on victims to report crimes and the criminal justice system to respond to their reports (Blumstein & Larson, 1971). Research suggests that there is a large “dark figure” for stalking. For example, in the United States, victimization surveys indicate that the rate of stalking reported by community residents is much higher than the rates of stalking arrest, charge, or conviction (Brewster, 2000; Jordan et al., 2003; Logan et al., 2009). This is consistent with evidence that one of the most common tactics used by law enforcement to investigate and respond to reports of stalking is to issue a formal warning to the alleged perpetrator to encourage desistence. For example, Storey and Hart (2011) reported in a study of cases referred to the anti-stalking unit of a municipal police force that formal warnings were issued in 59% of cases and were the final investigation or response tactic used in 29% of cases. In our opinion, the optimal strategy for defining recidivism is to use as broad a definition as possible (in light of the data being collected or coded) and the optimal strategy for analyzing recidivism is to use multiple nested definitions. This approach will facilitate the comparison of findings across studies. Research should also try to code and analyze specific aspects of stalking recidivism to study such things as whether the recidivism targeted the same or new victims (i.e., persistent versus serial stalking).

## Conclusion

The findings of the current study add to the growing body of literature on risk for stalking recidivism and also provide further support for the utility of the SAM for stalking risk assessment. Despite this, our understanding of stalking recidivism and how best to forecast it is limited. The diversity and complexity of stalking behavior, perpetrators, and defining recidivism present challenges that are certainly daunting, but by no means insurmountable. Resolving and clarifying the above issues would contribute substantially to the violence risk assessment field’s understanding of stalking behavior. The field eagerly awaits more research.

## Supplemental Material

sj-docx-1-asm-10.1177_10731911221086050 – Supplemental material for Forecasting Stalking Recidivism Using the Guidelines for Stalking Assessment and Management (SAM) Supplemental material, sj-docx-1-asm-10.1177_10731911221086050 for Forecasting Stalking Recidivism Using the Guidelines for Stalking Assessment and Management (SAM) by Sarah H. Coupland, Jennifer E. Storey, P. Randall Kropp and Stephen D. Hart in Assessment
