# Supplementary material for: Forecasting Stalking Recidivism Using the Guidelines for Stalking Assessment and Management (SAM)
Source: Assessment. 2022 Apr 18;30(4):1168–81. doi: 10.1177/10731911221086050 (PMC10149883; doi:10.1177/10731911221086050)
Supplement: sj-docx-1-asm-10.1177_10731911221086050 – Supplemental material for Forecasting Stalking Recidivism Using the Guidelines for Stalking Assessment and Management (SAM) [file sj-docx-1-asm-10.1177_10731911221086050.docx]

**Online Supplemental Materials**

|  | Recent | | | | |  |  | Past | | | | | | |  |
| --- | --- | --- | --- | --- | --- | --- | --- | --- | --- | --- | --- | --- | --- | --- | --- |
| SAM Domain/Factor | N | P | Y |  | AC_2_ | [95% CI] |  | | N | P | Y |  | AC_2_ | [95% CI] | |
| Nature of Stalking |  |  |  |  |  |  |  | |  |  |  |  |  |  | |
| N1 Communicates about victim | 49% | 10% | 41% |  | .75 | [.63, .87] |  | | 84% | 1% | 15% |  | .87 | [.75, 1.00] | |
| N2 Communicates with victim | 14% | 2% | 84% |  | .92 | [.82, 1.00] |  | | 72% | 2% | 26% |  | .85 | [.71, 1.00] | |
| N3 Approaches victim | 25% | 3% | 72% |  | .83 | [.73, .93] |  | | 76% | 2% | 22% |  | .85 | [.71, .99] | |
| N4 Direct contact with victim | 31% | 5% | 64% |  | .75 | [.59, .91] |  | | 78% | 1% | 21% |  | .86 | [.65, 1.00] | |
| N5 Intimidates victim | 8% | 14% | 78% |  | .85 | [.74, .94] |  | | 69% | 2% | 29% |  | .78 | [.55, 1.00] | |
| N6 Threatens victim | 43% | 8% | 49% |  | .76 | [.53, .99] |  | | 79% | 2% | 19% |  | .87 | [.71, 1.00] | |
| N7 Physically violent toward victim | 73% | 8% | 19% |  | .84 | [.71, 98] |  | | 85% | 2% | 13% |  | .85 | [.68, 1.00] | |
| N8 Stalking is persistent | 14% | 30% | 56% |  | .74 | [.48, .99] |  | | 75% | 10% | 15% |  | .85 | [.67, 1.00] | |
| N9 Stalking is escalating | 20% | 33% | 47% |  | .56 | [.39, .74] |  | | 78% | 8% | 14% |  | .86 | [.70, 1.00] | |
| N10 Stalking involves supervision violations | 37% | 11% | 52% |  | .75 | [.62, .88] |  | | 83% | 1% | 16% |  | .86 | [.70, 1.00] | |

Table OSM1

*Distribution of Consensus Presence Ratings (Recent and Past) for and Interrater Reliability of Individual SAM Factors*

*table continues...*

|  | Recent | | | | |  |  | Past | | | | | | |  |
| --- | --- | --- | --- | --- | --- | --- | --- | --- | --- | --- | --- | --- | --- | --- | --- |
| SAM Domain/Factor | N | P | Y |  | AC_2_ | [95% CI] |  | | N | P | Y |  | AC_2_ | [95% CI] | |
| Perpetrator Risk Factors |  |  |  |  |  |  |  | |  |  |  |  |  |  | |
| P1 Angry | 13% | 19% | 68% |  | .83 | [.73, .93] |  | | 39% | 14% | 47% |  | .63 | [.43, .84] | |
| P2 Obsessed | 13% | 23$ | 64% |  | .85 | [.78, .92] |  | | 64% | 10% | 26% |  | .75 | [.49, 1.00] | |
| P3 Irrational | 45% | 18% | 37% |  | .80 | [.70, .91] |  | | 75% | 6% | 19% |  | .84 | [.74, .93] | |
| P4 Unrepentant | 8% | 25% | 67% |  | .82 | [.72, 92] |  | | 53% | 8% | 39% |  | .66 | [.36, .97] | |
| P5 Antisocial lifestyle | 31% | 31% | 38% |  | .77 | [.58, .95] |  | | 51% | 17% | 32% |  | .79 | [.63, .96] | |
| P6 Intimate relationship problems | 13% | 7% | 80% |  | .92 | [.81, .99] |  | | 47% | 12% | 41% |  | .79 | [.63, .96] | |
| P7 Non-intimate relationships problems | 27% | 37% | 36% |  | .63 | [.48, .78] |  | | 54% | 20% | 26% |  | .71 | [.60, .82] | |
| P8 Distressed | 44% | 32% | 24% |  | .62 | [.36, .88] |  | | 57% | 16% | 27% |  | .67 | [.48, .85] | |
| P9 Substance use problems | 53% | 18% | 29% |  | .75 | [.47, 1.00] |  | | 50% | 6% | 44% |  | .67 | [.48, .85] | |
| P10 Employment and financial problems | 14% | 20% | 68% |  | .84 | [.76, .92] |  | | 46% | 17% | 37% |  | .61 | [.45, .77] | |

*table continues...*

|  | Recent | | | | |  |  | Past | | | | | | |  |
| --- | --- | --- | --- | --- | --- | --- | --- | --- | --- | --- | --- | --- | --- | --- | --- |
| SAM Domain/Factor | N | P | Y |  | AC_2_ | [95% CI] |  | | N | P | Y |  | AC_2_ | [95% CI] | |
| Victim Vulnerability Factors |  |  |  |  |  |  |  | |  |  |  |  |  |  | |
| V1 Inconsistent behavior toward perpetrator | 58% | 19% | 23% |  | .78 | [.60, .95] |  | | 69% | 10% | 21% |  | .78 | [.60, .97] | |
| V2 Inconsistent attitude toward perpetrator | 72% | 17% | 11% |  | .82 | [.74, .98] |  | | 82% | 7% | 11% |  | .86 | [.74, .97] | |
| V3 Inadequate access to resources | 88% | 7% | 5% |  | .90 | [.83, .98] |  | | 97% | 3% | 0% |  | .94 | [.84, 1.00] | |
| V4 Unsafe living situation | 37% | 35% | 28% |  | .56 | [.38, .74] |  | | 75% | 5% | 20% |  | .80 | [.61, 1.00] | |
| V5 Problems caring for dependents | 65% | 19% | 16% |  | .81 | [.61, 1.00] |  | | 89% | 1% | 10% |  | .88 | [.66, 1.00] | |
| V6 Intimate relationship problems | 53% | 15% | 32% |  | .81 | [.57, 1.00] |  | | 80% | 3% | 17% |  | .79 | [.60, .99] | |
| V7 Non-intimate relationship problems | 83% | 9% | 8% |  | .89 | [.82, .97] |  | | 92% | 3% | 5% |  | .92 | [.79, 1.00] | |
| V8 Distressed | 54% | 32% | 14% |  | .72 | [.55, .93] |  | | 84% | 6% | 10% |  | .89 | [.74, 1.00] | |
| V9 Substance use problems | 92% | 7% | 1% |  | .93 | [.86, .99] |  | | 95% | 2% | 3% |  | .95 | [.85, 1.00] | |
| V10 Employment and financial problems | 74% | 22% | 4% |  | .87 | [.73, 1.00] |  | | 96% | 4% | 0% |  | .94 | [.82, 1.00] | |

*Note*. *N* = 100. SAM = Guidelines for Stalking Assessment and Management; N = *No/Absent*; P = *Possibly/Partially Present*; Y = *Yes/Present*; Ordinal weights and unconditional standard errors applied to coefficient calculations. Qualitative classifications are described using the Landis and Koch (1977) approach.
